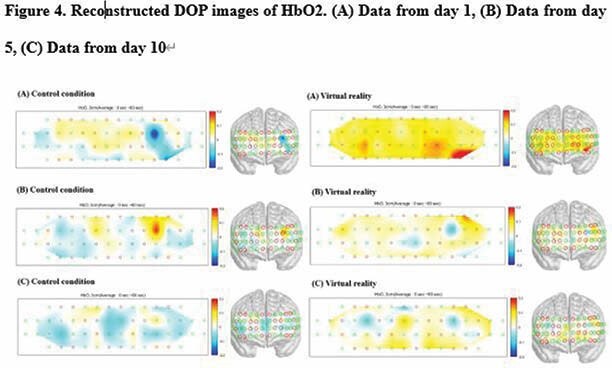# 70 Effect of of Virtual Reality on Pain Reduction in Robot Training in Burn Patients

**DOI:** 10.1093/jbcr/irac012.073

**Published:** 2022-03-23

**Authors:** So Young Joo, Cheong Hoon Seo

**Affiliations:** Hangang Sacred Heart Hospital, Yeongdeungpo-gu, Seoul-t'ukpyolsi; Hallym University, Seoul, Seoul-t'ukpyolsi

## Abstract

**Introduction:**

Burn injuries and their treatment are extremely painful. This study aimed to determine whether virtual reality (VR) can reduce pain during robot-assisted gait training (RAGT) in burn patients, by analyzing the cerebral blood flow (CBF) in the prefrontal cortex over time, using functional near-infrared spectroscopy (fNIRS).

**Methods:**

The patients included in this study complained of a pain score≥5 on a visual analog scale (VAS) during RAGT, which was performed 10 times for 2 weeks. Each session consisted of 15 min of VR application, a 2 min break, and 15 min without VR. The average values of oxy-hemoglobin and deoxy-hemoglobin in the prefrontal cortex using fNIRS were calculated at four stages: temporal delay time with only RAGT, RAGT without VR, temporal delay time with RAGT and VR, and RAGT with VR. The pain scores and CBF were evaluated in sessions 1, 5, and 10 of the RAGT.

**Results:**

The mean VAS pain scores were significantly lower (P< 0.05) in the experimental condition than in the control condition. Oxy-hemoglobin in the prefrontal lobe increased significantly when RAGT was performed with VR.

**Conclusions:**

Therefore, VR may be a strong non-pharmacological pain reduction technique for burn patients during physical therapy.